# Post-pacing interval after failed anti-tachycardia pacing for scar-related ventricular tachycardia: implications for the location of the critical substrate

**DOI:** 10.1093/europace/euaf069

**Published:** 2025-05-09

**Authors:** Masafumi Sugawara, Jakub Sroubek, Justin Lee, Shady Nakhla, Oussama Wazni, Pasquale Santangeli, Koji Higuchi

**Affiliations:** Department of Cardiovascular Medicine, Cardiac Electrophysiology and Pacing Section, Cleveland Clinic, 9500 Euclid Avenue, Cleveland, OH 44195, USA; Department of Cardiovascular Medicine, Cardiac Electrophysiology and Pacing Section, Cleveland Clinic, 9500 Euclid Avenue, Cleveland, OH 44195, USA; Department of Cardiovascular Medicine, Cardiac Electrophysiology and Pacing Section, Cleveland Clinic, 9500 Euclid Avenue, Cleveland, OH 44195, USA; Department of Cardiovascular Medicine, Cardiac Electrophysiology and Pacing Section, Cleveland Clinic, 9500 Euclid Avenue, Cleveland, OH 44195, USA; Department of Cardiovascular Medicine, Cardiac Electrophysiology and Pacing Section, Cleveland Clinic, 9500 Euclid Avenue, Cleveland, OH 44195, USA; Department of Cardiovascular Medicine, Cardiac Electrophysiology and Pacing Section, Cleveland Clinic, 9500 Euclid Avenue, Cleveland, OH 44195, USA; Department of Cardiovascular Medicine, Cardiac Electrophysiology and Pacing Section, Cleveland Clinic, 9500 Euclid Avenue, Cleveland, OH 44195, USA

**Keywords:** ICD/CRT-D, Failed ATP, PPI−TCL, Septal intramural VT

## Introduction

Anti-tachycardia pacing (ATP) is a sequential pacing stimulus delivered from an implantable cardioverter defibrillator (ICD) to terminate reentrant ventricular tachycardia (VT). If ATP successfully entrains VT but fails to terminate, it can be considered as an entrainment pacing maneuver from the right ventricle (RV) apical septum, where the ICD lead is normally placed.^1,2^ In this regard, the post-pacing interval—tachycardia cycle length (PPI−TCL) after failed ATP may correlate with the distance from the RV apical septum to the substrate of scar-related VT and may provide diagnostic value similar to the conventional entrainment pacing, though that remains unexplored. Furthermore, septal VT with intramural substrate, which predominantly occurs in non-ischemic cardiomyopathy (NICM),^2^ was separately identified in this study to evaluate the difference from septal endocardial VT.

## Methods

This retrospective study analyzed 66 VT ablations performed between November 2021 and February 2024 in 64 patients with either ischemic cardiomyopathy (ICM) or NICM. All included patients had prior ICD or cardiac resynchronization therapy with defibrillator (CRT-D) implantation. Inclusion criteria were limited to patients with failed ATP prior to VT ablation and RV ICD leads positioned at the apical septum. During the ablation procedure, intracardiac electrograms (EGMs) of induced VTs were compared with the stored ICD-EGMs to identify the clinical VT.^3^ The reentrant mechanism of each VT was intra-procedurally confirmed based on the activation pattern in the 3D mapping and the response to the conventional entrainment pacing.

### Retrospective analysis of EGM and VT substrate location

**Figure 1:**
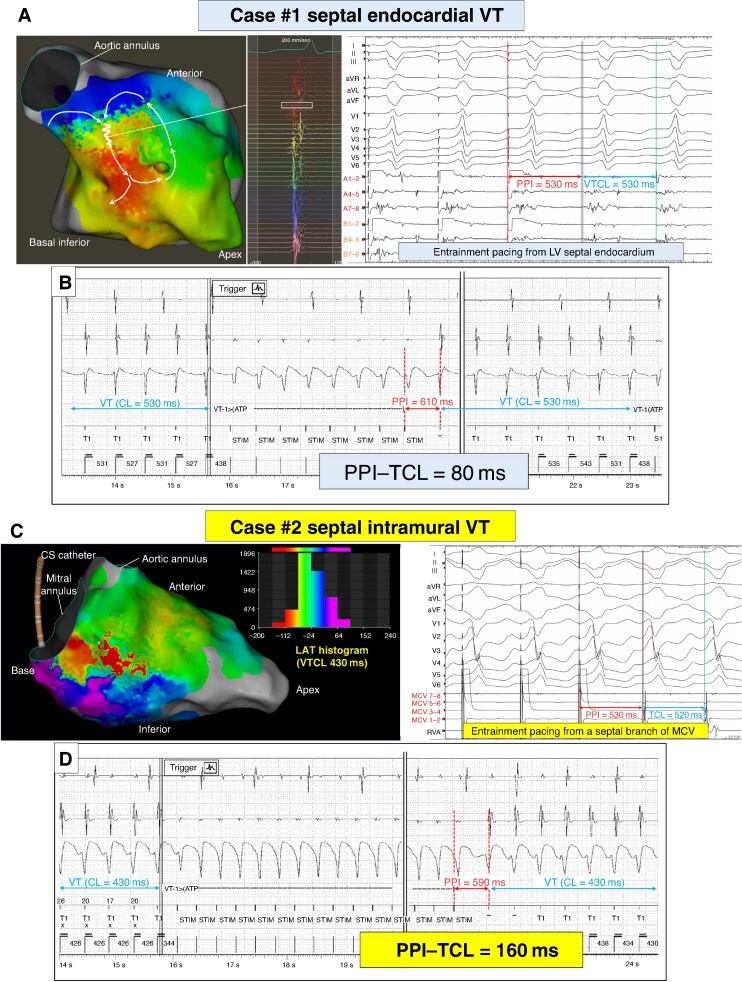
(*A*) Activation mapping and EGM of septal endocardial VT of NICM (case #1). An entire reentrant VT circuit was depicted in LV basal anteroseptal region, and a long-fractionated potential was recorded, which was suggestive of VT critical isthmus. At this site, VT with 530 ms of TCL was entrained by pacing with 500 ms of pacing CL from multielectrode catheter (bipolar A1–2) and concealed entrainment with short PPI−TCL was obtained. Endocardial RF ablation at this site successfully terminated this VT. (*B*) EGM of failed ATP recorded by ICD before VT ablation in Case#1. The clinical VT with 530 ms of TCL exhibited overdrive suppression by a burst ATP with pacing CL of 440 ms (83% of TCL). PPI after failed ATP was 610 ms and PPI−TCL value was 80 ms. (*C*) Activation mapping and EGM of septal intramural VT of NICM (Case #2). An entire VT circuit was uncertain and the LAT histogram showed that nearly half of the cycle length was missing. Endocardial RF ablation at LV basal infero-septum slowed down the CL of clinical VT (430 to 520 ms), but did not terminate. Entrainment pacing from a multielectrode catheter placed in a branch of MCV (bipolar 3–4) exhibited concealed entrainment with short PPI−TCL (10 ms) indicating an intramural substrate being involved in this reentrant VT. Eventually, ethanol injection followed by double-balloon technique in the septal branch of MCV successfully terminated and abolished this VT. (*D*) EGM of failed ATP recorded by ICD before VT ablation in Case#2. The clinical VT with 430 ms of TCL exhibited overdrive suppression by a burst ATP with a pacing CL of 360 ms (83% of TCL). PPI after failed ATP was 590 ms and the PPI−TCL value was 160 ms, which was significantly longer than the PPI−TCL after failed ATP in Case#1, despite the similar distance from the site of ATP (RV apex). ATP, anti-tachycardia pacing; CS; coronary sinus; EGM, intracardiac electrogram; ICD, implantable cardioverter defibrillator; ICM, ischemic cardiomyopathy; LAT, local activation time; LV, left ventricle; MCV, mid-cardiac vein; NICM, non-ischemic cardiomyopathy; PPI, post-pacing interval; RF, radiofrequency; RV, right ventricle; TCL, tachycardia cycle length; VT, ventricular tachycardia.

All EGMs recorded within 24 months prior to the index VT ablation were reviewed. The clinical VT was identified as the most dominant VT, determined based on the TCL and the morphology of the EGMs. PPI was defined as the time interval between the last pacing spike of ATP and the first beat of resumed VT (*Figure1B* and *D*).^4^

The locations of the critical VT substrate were retrospectively identified based on substrate mapping and VT activation mapping, which included delayed or abnormal potential in substrate mapping with excellent pace mapping for unstable VTs. For stable VTs, critical sites were determined by diastolic components during VT activation mapping with concealed entrainment with short PPI−TCL (<30 ms). In all cases, the targeted critical sites had to demonstrate successful abolition of clinical VT. During this review process of 3D mapping data, VT with unidentified critical sites, failed ablation procedure, VTs associated with broad extensive scar, non-reentrant VTs, and fascicular VTs were excluded.

### Definition of septal intramural VT

To evaluate the influence of septal intramural substrate, septal VTs were further categorized into endocardial and intramural groups. Septal intramural VT was determined based on the following criteria.^5,6^

1. Relatively early activation at the interventricular septum in endocardial mapping.
2. Absence of diastolic/pre-systolic activation, concealed entrainment with shorter PPI−TCL (<30 ms), and excellent pace map with ≥90% match in the endocardial mapping.
3. The earlier activation time recorded in the septal perforator vein compared with the septal endocardium.
4. Abolition of clinical VT requiring sequential unipolar ablation both from left ventricle (LV) and RV septum.
5. Abolition of clinical VT requiring ethanol injection into septal perforator vein.

In this study, the presence of (1) and (2), along with at least one of criteria (3), (4), or (5) was considered as septal intramural VT.

### Statistical analysis

Continuous and categorical variables were expressed as mean ± standard deviation or median (full range) and numbers [percentage (%)], respectively. The PPI−TCL in each group were compared using ANOVA and Bonferroni correction, and the corrected *P*-value of <0.0033 was considered statistically significant. The PPI−TCL values between LV-septal endocardial and septal intramural VT were compared using the Mann–Whitney *U* test. The best cut-off value of PPI−TCL to differentiate them was evaluated by the receiver operating characteristic curve. JMP version 16.2.0 (SAS Institute) was utilized for statistical analysis.

## Results

### Localisation of VT substrates

Among 66 VTs, seven were determined as septal intramural VTs based on pre-systolic potential recorded in an intramural coronary venous branch (*n* = 4), the requirement of sequential unipolar RF ablation from biventricular septum (*n* = 4), and the requirement of ethanol injection into septal perforator vein (*n* = 4). The remaining 59 VTs were categorized into five other groups: RV (*n* = 6), LV-septal endocardial (*n* = 28), LV-inferior (*n* = 8), LV-lateral (*n* = 12), and LV-anterior VT (*n* = 5). Two patients with NICM each had two distinct clinical VTs and underwent VT ablations for each VT at separate time points. One patient had RV-VT and LV-anterior VT, while the other had LV-lateral VT and septal intramural VT. Patient characteristics, including the number of cases with VT termination during ablation, concealed entrainment, diastolic potential during VT, and excellent pacemap, are summarized in *Table 1*.

**Table 1:** Patient characteristics at the time of index VT ablation

	Total	RV-VT	LV-septal	LV-inferior VT	LV-lateral VT	LV-anterior VT	Septal-intramural VT	*P-*value^a^
	(*n* = 66)	(*n* = 6)	endocardial VT	(*n* = 8)	(*n* = 12)	(*n* = 5)	(*n* = 7)	
			(*n* = 28)					
Patient characteristics								
Age, years	68.0 ± 8.9	64.3 ± 3.8	70.3 ± 6.5 ^b^	70.1 ± 6.8	67.8 ± 13.1	57.6 ± 13.2	72.1 ± 5.7	0.045
Male gender, *n*	57 (86)	5 (83)	24 (86)	6 (75)	11 (92)	5 (100)	6 (86)	0.764
Body mass index, kg/m^2^	29.3 ± 6.5	30.8 ± 7.3	28.8 ± 6.1	28.7 ± 6.5	29.1 ± 8.1	31.4 ± 5.9	29.3 ± 6.6	0.929
Left ventricular ejection fraction, %	31.2 ± 11.4	33.0 ± 13.5	29.3 ± 11.6	33.9 ± 9.0	34.3 ± 12.5	24.4 ± 9.5	33.4 ± 10.9	0.448
Non-ischemic cardiomyopathy, *n*	31 (47)	5 (83)	11 (39)	1 (13)	6 (50)	2 (40)	6 (86)	0.064
History of prior VT ablation, *n*	21 (32)	2 (33)	9 (32)	1 (13)	4 (33)	0 (0)	5 (71)	0.154
History of cardiac surgery, *n*	27 (41)	1 (17)	10 (36)	5 (62)	7 (58)	3 (60)	1 (4)	0.073
Device								
Single chamber ICD, *n*	10 (15)	0 (0)	4 (14)	1 (13)	3 (25)	0 (0)	2 (29)	0.383
Dual chamber ICD, *n*	27 (41)	3 (50)	9 (32)	4 (50)	5 (42)	3 (60)	3 (43)	0.829
CRT-D, *n*	29 (44)	3 (50)	15 (54)	3 (38)	4 (33)	2 (40)	2 (29)	0.767
Medications								
Beta blocker, *n*	56 (85)	4 (67)	24 (86)	6 (75)	11 (92)	4 (80)	7 (100)	0.459
Amiodarone, *n*	46 (67)	3 (50)	19 (68)	5 (63)	9 (75)	5 (100)	5 (71)	0.412
Mexiletine, *n*	26 (39)	0 (0)	11 (39)	4 (50)	5 (42)	2 (40)	0 (0)	0.199
Sotalol, *n*	5 (8)	2 (33)	2 (7)	0 (0)	1 (8)	0 (0)	0 (0)	0.262
Dofetilide, *n*	1 (2)	0 (0)	0 (0)	1 (13)	0 (0)	0 (0)	0 (0)	0.502
Total number of AADs, *n*	2.0 (0–4.0)	1.5 (1.0–2.0)	2.0 (0–4.0)	2.0 (1.0–3.0)	2.0 (1.0–3.0)	2.0 (2.0–3.0)	2.0 (1.0–2.0)	0.512
Clinical VT characteristics								
Morphology (RBBB/LBBB), *n*	43/22	0/6	16/12	7/1	11/1	3/2	6/1	—
Axis (superior/inferior), *n*	37/29	3/3	17/11	7/1	7/5	1/4	2/5	—
Axis (right/left), *n*	35/31	1/5	13/15	4/4	9/3	4/1	4/3	—
Determinants of VT substrate location								
Endocardial VT groups								
RFA site leading to VT termination, *n*	—	3 (50)	18 (64)	3 (38)	9 (75)	5 (100)	—	—
Concealed entrainment, *n*	—	3 (50)	11 (39)	3 (38)	6 (50)	3 (60)	—	—
Diastolic potential during VT, *n*	—	4 (67)	23 (82)	6 (75)	10 (83)	3 (60)	—	—
Excellent pace map, *n*	—	3 (50)	6 (21)	2 (25)	4 (33)	0 (0)	—	—
Intramural VT group								
Coronary venous mapping, *n*	—	—	—	—	—	—	4 (57)	—
Sequential unipolar ablation, *n*	—	—	—	—	—	—	4 (57)	—
Ethanol injection to CS branch, *n*	—	—	—	—	—	—	4 (57)	—
EGM analysis								
Follow-up period, month	2.5 (1.0–24.0)	10.5 (1.0–19.0)	6.0 (1.0–24.0)	2.0 (1.0–20.0)	1.5 (1.0–24.0)	1.0 (1.0–8.0)	4.0 (1.0–13.0)	0.642
Number of device interrogation, *n*	4.0 (1.0–28.0)	9.5 (1.0–18.0)	3.5 (1.0–28.0)	2.0 (1.0–14.0)	3.5 (1.0–13.0)	2.0 (1.0–23.0)	7.0 (1.0–13.0)	0.904
Number of ATP EGMs, *n*	13.0 (1.0–73.0)	8.5 (5.0–13.0)	28.5 (2.0–73.0) ^c^	9.5 (1.0–36.0)	10.0 (1.0–21.0)	14.0 (7.0–50.0)	12.0 (3.0–44.0)	0.019
Number of eligible failed ATP EGMs, *n*	3.0 (1.0–48.0)	2.5 (1.0–9.0)	8.5 (1.0–48.0)	1.5 (1.0–15.0)	2.0 (1.0–11.0)	5.0 (1.0–17.0)	4.0 (1.0–8.0)	0.066
Mean TCL of VT, ms	407 ± 71	341 ± 71	415 ± 71	411 ± 65	391 ± 57	452 ± 49	332 ± 36	0.372
Mean ATP cycle length, ms	333 ± 61	286 ± 64	340 ± 54	335 ± 52	313 ± 46	360 ± 41	281 ± 29	0.407
Mean PPI−TCL, ms	139 ± 69	75 ± 39 ^d^	119 ± 61 ^e^	162 ± 23	177 ± 27	219 ± 49	233 ± 71	<0.0001

Continuous variables are represented as mean ± standard deviation or median (full range). Categorical variables are represented as number (%).

^a^ *P*-values represent the results of statistical analysis using ANOVA for continuous variables and Fisher’s exact test for categorical variables, with a *P*-value < 0.05 as a statistical significance. A post-hoc analysis is performed using Bonferroni correction with a *P*-value < 0.0033 as a statistical significance considering the number of groups (6 groups).

^b^Mean age of LV-septal endocardial VT group is higher than that of LV-anterior VT group (*P* = 0.0029).

^c^Median number of ATP EGMs of LV-septal endocardial VT group is higher than that of LV-lateral VT group (*P* = 0.0029).

^d^Mean PPI−TCL of RV-VT group is shorter than those of LV-lateral VT group (*P* < 0.0001), LV-anterior VT group (*P* < 0.0001) and septal intramural VT group (*P* < 0.0001).

^e^Mean PPI−TCL of LV-septal endocardial VT group is shorter than those of LV-lateral VT group (*P* < 0.0001), LV-anterior VT group (*P* = 0.0003) and septal intramural VT group (*P* < 0.0001).

AADs, anti-arrhythmic drugs; ATP, anti-tachycardia pacing; CRT-D, cardiac resynchronization therapy with defibrillator; CS, coronary sinus; EGM, intracardiac electrogram; ICD, implantable cardioverter defibrillator; LBBB, left bundle branch block; LV, left ventricle; PPI, post-pacing interval; RBBB, right bundle branch block; RFA, radiofrequency ablation; RV, right ventricle; TCL, tachycardia cycle length; VT, ventricular tachycardia.

### Relationship between the location of VT substrate and PPI−TCL after failed ATP

Among the 66 clinical VTs, a median of 3.0 (1.0–47.0) eligible failed ATP EGMs were identified and analyzed per patient over a median of 2.5 (1.0–24.0) months before each index VT ablation procedure. The mean TCL of these VTs was 407 ± 71 ms, while the mean ATP cycle length was 333 ± 55 ms. There was no statistical difference between the mean TCL of VT and the ATP cycle length among the six groups. The PPI−TCL increased in correlation with the estimated distance from the pacing site (RV apex) to the VT substrate location (*Table 1*). Notably, however, the septal intramural VT group exhibited the longest PPI−TCL despite its anatomical proximity to the pacing site. The mean PPI−TCL for septal intramural VTs (*n* = 7) was significantly longer than that of LV-septal endocardial VTs (*n* = 28) (233 ± 71 ms vs. 119 ± 61 ms, *P* = 0.001), despite their similar location of origin. The best cut-off value of PPI−TCL >150 ms favoured septal-intramural VT with the area under curve of 0.90 (sensitivity = 82%; specificity = 100%). Examples of LV-septal endocardial and intramural VTs are shown in *Figure 1A–D*.

## Discussion

To the best of our knowledge, this retrospective study is the first to report the relationship between PPI−TCL after failed ATP and the location of reentrant VT substrate, showing similarities to the conventional entrainment pacing.^1^ Notably, however, septal VT with an intramural substrate exhibited significantly longer PPI−TCL than septal endocardial VTs. A plausible explanation is that additional time is required for the paced wavefront to longitudinally traverse the entire substrate at the deep intraventricular septum and return to the pacing site, resulting in prolonged PPI−TCL compared with VTs confined to an endocardial substrate. One of the major limitations is that the cycle length of ATP is typically shorter than conventional entrainment pacing (80–88% of TCL).^7^ At a faster pacing rate, the refractory period of the tachycardia circuit might be infringed, resulting in inflated PPI−TCL values.^8^ However, in this study, the ATP cycle length was evenly distributed across groups (*Table 1*), suggesting that the observed differences in PPI−TCL more likely reflect the substrate’s distance from the pacing site. Other limitations can be as follows: (i) The true VT mechanism cannot be fully determined by ATP alone. However, we confirmed the reentrant mechanism intra-procedurally using conventional electrophysiological study and 3D mapping. (ii) Although overdrive suppression of VT was confirmed in all EGMs and only reentrant VTs were included, it remains uncertain whether all ATP episodes fully ‘entrained’ the VT. (iii) The location of the RV lead may have varied slightly between cases. (iv) The use of anti-arrhythmic drugs was not standardized across patients, which may have impacted PPI−TCL. (v) The mean TCL of VTs in this cohort was relatively slow. Additional studies are needed to assess whether these findings apply to faster VTs, which are less likely to be captured or reset by ATP.

## Conclusion

The PPI−TCL after failed ATP implied the correlation with the estimated distance from the RV-apical septum to the clinical VT substrate. The PPI−TCL of septal intramural VT was significantly longer than that of septal endocardial VT, despite their similar anatomical proximity to the pacing site.

## Data Availability

The data underlying this article will be shared on reasonable request to the corresponding author.
