# Correction to: Post-pacing interval after failed anti-tachycardia pacing for scar-related ventricular tachycardia: implications for the location of the critical substrate

**DOI:** 10.1093/europace/euag106

**Published:** 2026-05-14

This is a correction to: Masafumi Sugawara, Jakub Sroubek, Justin Lee, Shady Nakhla, Oussama Wazni, Pasquale Santangeli, Koji Higuchi, Post-pacing interval after failed anti-tachycardia pacing for scar-related ventricular tachycardia: implications for the location of the critical substrate, *EP Europace*, Volume 27, Issue 5, May 2025, euaf069, https://doi.org/10.1093/europace/euaf069

In the originally published version of this manuscript, dates in Table 1 were given in ‘day/month’ format. This has been corrected to ‘month/day’ format.

